# Profile of patients in the psychiatric Convalescence Unit at Husa

**DOI:** 10.1192/j.eurpsy.2025.1798

**Published:** 2025-08-26

**Authors:** E. D. Alvarez, A. M. M. Sanchez, P. A. Olivera, M. L. Argudo, N. M. C. Espada, R. D. A. Gómez, J. I. D. L. I. Larrad, C. I. F. Velasco, A. Z. Gomez

**Affiliations:** 1 CAUSA, Salamanca, Spain

## Abstract

**Introduction:**

The Convalescence Unit provides semi-open inpatient care during the subacute phases of psychiatric illness, with the goal of restructuring and reorganizing the personality after a crisis or relapse.

**Objectives:**

The main objective is to observe the profile of patients admitted to the Convalescence Unit who could benefit from this type of resource.

**Methods:**

A retrospective, observational study was conducted by collecting and analyzing data in 2019, which 61 patients were admitted in this resource. The profile of patients during the year 2019 was analyzed.

**Results:**

Of a total of 61 patients, 47 were referred from the Brief Hospitalization Unit (UHB), 11 from the Mental Health Team (ESM), and 3 from other facilities. By gender, there were 38 women and 23 men. The average age was 44.16 years. Regarding the type of admission, 29 were voluntary and 32 involuntary. Seventeen patients had been readmitted from previous years. The diagnoses were: 10 patients with bipolar disorder, 14 with schizophrenia, 6 with substance-induced psychosis, 2 with schizoaffective disorder, 10 with anxiety-depressive syndrome, 5 with dysthymia, 2 with obsessive-compulsive disorder, 7 with delusional disorder, and 6 with personality disorders. Of these patients, 58 received therapeutic discharge, 1 voluntary discharge, and 2 were transferred to other facilities. Regarding marital status, 43 were single, 7 married, 4 widowed, and 7 divorced. The average length of stay was 48.56 days.

**Conclusions:**

As for the profile of patients admitted to the Convalescence Unit, it is not possible to determine a statistically significant pattern. However, we observed that most are women with severe mental health disorders. Notably, the majority are single, suggesting less family support, and a high percentage are admitted involuntarily, often from the UHB, to extend their stay for the management of subacute conditions in a more controlled environment. This helps ensure adherence to treatment, which is both difficult and crucial for a favorable outcome in these patients.

**Disclosure of Interest:**

None Declared

